# Editorial: The legacy of Dr. Candance Pert: recent advances in the neuropeptides and neuroreceptors research

**DOI:** 10.3389/fnmol.2023.1295240

**Published:** 2023-10-03

**Authors:** Patrícia Zschaber Anacleto, Julliane V. Joviano-Santos

**Affiliations:** ^1^Post-Graduate Program in Health Sciences, Faculdade Ciências Médicas de Minas Gerais, Belo Horizonte, Minas Gerais, Brazil; ^2^Laboratório de Investigações NeuroCardíacas, Ciências Médicas de Minas Gerais (LINC CMMG), Belo Horizonte, Minas Gerais, Brazil

**Keywords:** neuropeptides, neuroreceptors, neurodegenerative disorders (ND), Dr. Candance Pert, neuroscience

Neurodegenerative disorders related to aging pose a significant global health challenge (Teleanu et al., 2022). These disorders involve the gradual deterioration of neuron structure or function, leading to a range of mental and physical consequences. This decline significantly impacts the quality of life and is ultimately fatal. The manifestations of neurodegenerative disorders encompass various mental symptoms such as changes in cognition, learning, and memory, as well as conditions like dementia, depression, and anxiety, along with movement-related issues (Liu et al., 2023).

Neuropeptides and neuroreceptors research could bring valuable tools for treating and studying several neurodegenerative disorders. In this regard, the neuroscience field commemorates the birthday of Dr. Candace Pert (1946–2013), a distinguished neuroscientist. While pursuing her graduate studies at the Johns Hopkins University School of Medicine during the early 1970s, Dr. Pert made a notable contribution by identifying for the first time an opioid receptor (Pert and Snyder, 1973, 1975). This pivotal revelation paved the way for extensive exploration into the role of neuropeptides in neurobiological information processing within the brain.

Recent advances in neuropeptides and neuroreceptors research could include targeted therapies focusing on understanding the role of specific neuropeptides and neuroreceptors in various neurological and psychiatric disorders. Improved techniques for mapping and imaging neuroreceptors' distribution and activity in the brain could provide deeper insights into their roles in various brain functions and disorders. Recent research may have uncovered the involvement of specific neuropeptides or neuroreceptors in the progression of neurodegenerative diseases like Alzheimer's, Parkinson's, or Huntington's disease (Cunnane et al., 2020). This could potentially open up new avenues for early detection and intervention.

Advances in understanding neuropeptides signaling might lead to better pain management strategies. Neuropeptides are known to play a crucial role in pain perception and modulation (Bán et al., 2020). Novel research might have elucidated the role of neuropeptides and neuroreceptors in mental health disorders such as depression, anxiety, schizophrenia, and bipolar disorder. For example, toxins from animals can display important pharmacological tools being a formidable toolkit for undertaking the treatments of neuronal conditions. In this Research Topic, we can observe, for instance, that some authors have recognized and validated the presence of five toxin groups that exhibit significant resemblances to unidentified secretory peptides found in mollusks, annelids, and ecdysozoans (Koch et al.). Animal toxins are products of natural selection and they can be found in a broad range of organisms (Yang et al., 2019). The analysis, purification, and synthesis of the components of different animal venoms' can be useful for the development of new pharmacological tools and drugs for a wide range of diseases, including neurodegenerative disorders. Although the data and experiments have been presented thoroughly, along with well-supported conclusions, certain inquiries still require attention. Neurotoxins, for instance, are substances that can cause harm or damage to the nervous system and hence a careful and clear consideration on study limitations are important to extrapolate the preclinical data.

Moreover, recent studies might have contributed to a better understanding of how neuropeptides and/or neuroreceptors influence synaptic plasticity, which is crucial for the development, learning and memory processes. Advances in research might have highlighted the role of them in neuroinflammatory processes, potentially providing insights into the mechanisms underlying neuroinflammatory diseases. New findings might have emerged regarding the role of neuropeptides and neuroreceptors in brain development, particularly during critical periods of growth and maturation. One example from our Topic is the significance of L-lactate in learning and memory which is substantial. Research involving rats revealed that introducing external L-lactate to the anterior cingulate cortex and hippocampus led to enhanced decision-making in the former and improved long-term memory formation in the latter. While the precise molecular mechanisms through which L-lactate delivers these advantageous outcomes are actively under scrutiny, this study uncovered the molecular signature from supplementing with L-lactate; that can trigger the upregulation of crucial controllers involved in mitochondrial biogenesis and the reinforcement of antioxidant defense (Akter et al.).

Finally, two other articles from our Research Topic that can be mentioned is a new molecular discovery regarding cells from the cerebellum. It was described a novel molecular marker, Pou3f1, that delineates specific sets of glutamatergic cerebellar nuclear neurons (Wu et al.). Of greater significance, the published study expands the shared understanding within the field regarding the molecular variety present in cerebellar cells. The second paper, aimed to explore the involvement of microglia in obsessive-compulsive disorder. It was created a pharmacological mouse model using the serotonin (5-HT) 1A/1B receptor agonist RU24969 to replicate the monoamine dysregulation observed in this condition. The authors assessed the changes in morphology and functionality of microglia within this model and they suggested new evidence points to a strong connection between microglial dysfunction and the manifestation of stereotypic behaviors. In their model, the Brain-derived neurotrophic factor (BDNF) might serve as a viable treatment approach for addressing these behaviors (Luo et al.).

In summary, studying neuroreceptors and bringing new Research Topics like ours in honor of Dr. Candance Pert is pivotal for advancing our understanding of the nervous system's function, dysfunction, and potential therapeutic interventions across a wide spectrum of neurological and psychiatric conditions. Many pharmaceutical drugs, including those used to treat neurological and psychiatric disorders, target specific neuroreceptors. Understanding the structure and function of neuroreceptors assists in designing drugs with more effective physicochemical and biological properties. Importantly, as our understanding of neuroreceptors advances, personalized treatment approaches can be developed based on an individual's receptor profile. This can lead to more tailored and effective treatments for various neurological conditions.

## Author contributions

PZ: Writing—review and editing. JJ-S: Conceptualization, Writing—original draft, Writing—review and editing.
